# An Approach for Cognitive Evaluation and Management of Grade 5 Arteriovenous Malformation-Associated Thalamic Dementia: A Case Report and Literature Review

**DOI:** 10.7759/cureus.39913

**Published:** 2023-06-03

**Authors:** Gunjanpreet Kaur, Karthik Narayanan, Kyle Schroeder, Firas Al Shakarchi, Abdullah M Hakoun

**Affiliations:** 1 Department of Neurology, Saint Louis University School of Medicine, St. Louis, USA

**Keywords:** vascular shunt, endovascular and surgical therapy, rapid dementia, bilateral thalamic, arteriovenous malformations

## Abstract

The clinical approach to managing high-grade arteriovenous malformations (AVMs) has been challenging due to its various presentations, surgical risk of complications, and impact on patients’ quality of life. We report a case of a 57-year-old female who experienced recurrent seizures and progressive cognitive decline secondary to a grade 5 cerebellar AVM. We reviewed the patient’s presentation and clinical course. We also searched the literature for studies, reviews, and case reports involving the management of high-grade AVMs. We outline our recommendations on how to approach these cases after a review of the currently available treatment options.

## Introduction

Arteriovenous malformation (AVM) is an abnormal tangle of low-resistance channels between arteries and veins, which disrupts normal blood flow and oxygen circulation. Interruption of blood flow can present as several clinical manifestations. AVMs generally affect a specific anatomical territory and seldom have holocephalic involvement. Clinical manifestations of AVMs usually present between the second and fourth decades of life. The most commonly reported symptoms include headaches, seizures, paralysis, numbness, and movement disorders [1]. Management of AVMs is dependent on presenting symptoms and surgical candidacy. Most symptomatic AVMs are surgically excised; however, surgical intervention may pose an elevated risk of complications and mortality in larger AVMs. Despite recent improvements in surgical techniques, evidence regarding the management of high-grade AVMs, defined as Spetzler-Martin (SM) grades 4 and 5, is lacking. In this case, we report rapid cognitive decline in a middle-aged patient secondary to grade 5 AVM with bilateral thalamic lesions and we review the most recent literature concerning the management of high-grade AVMs.

## Case presentation

**Figure 1:**
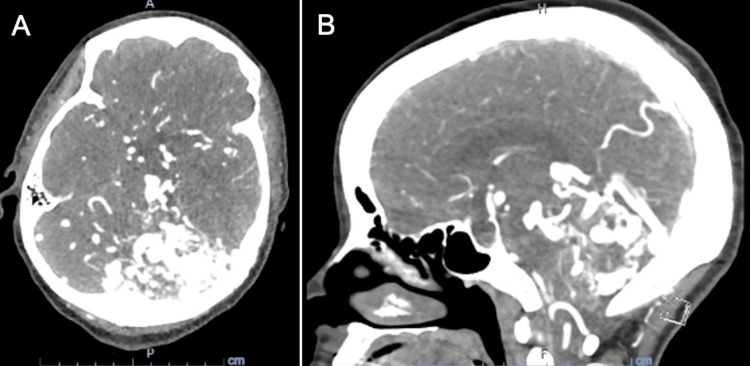
CT angiogram of the arteriovenous malformation (transverse (A) and sagittal (B) views) showing multiple arterial feeders seen from the left posterior inferior cerebellar artery and left superior cerebellar artery with dilation of the vertebral basilar system and bilateral internal carotid arteries, particularly on the left.

**Figure 2:**
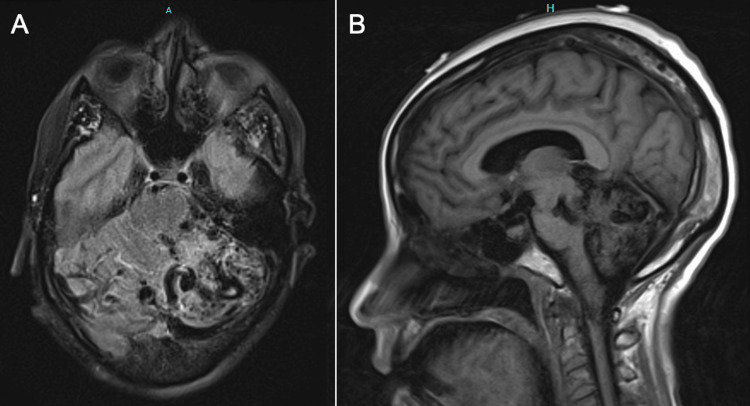
MRI of the brain FLAIR sequence (transverse (A) and sagittal (B) views) highlighting the significant atrophy in the cerebellum and showing a hypodensity in the left thalamus. FLAIR: fluid-attenuated inversion recovery.

A 57-year-old right-handed African American female was brought to our hospital after an episode of loss of consciousness. Upon arrival, she was noted to have right-sided flexion, left-sided extension, and forced gaze deviation to the left. Seizure-like activity was suspected, and she was administered 6 mg of intravenous lorazepam along with a 60 mg/kg load of levetiracetam, which resolved her symptoms. Computed tomography angiography (CTA) of the head revealed a vascular malformation involving the posterior fossa with a hyperattenuating density seen in the right cerebellar hemisphere extending supratentorially (Figure 1). Magnetic resonance imaging (MRI) of the brain confirmed a large left cerebellar AVM with a nidus measuring approximately 4.1 x 4.0 x 2.4 cm in maximum transaxial and craniocaudal dimensions (Figure 2). Venous congestion was noted in multiple structures, including the torcula, vein of Galen, and left basal vein of Rosenthal, bilateral cerebellar hemispheres, bilateral thalami, and left frontal lobe.

**Figure 3:**
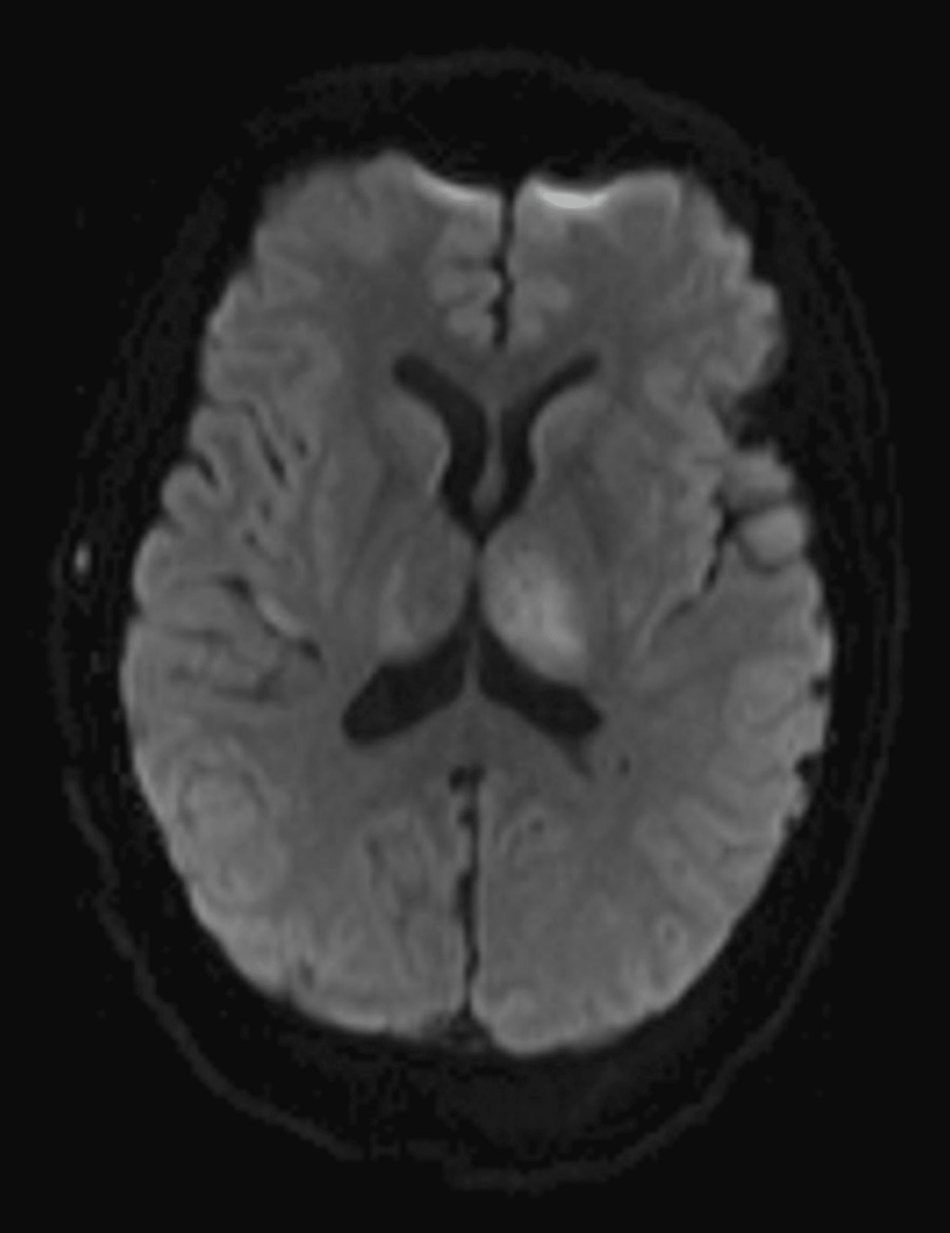
MRI of the brain DWI sequence in transverse view showing increased signal in bilateral thalamic structures, with the left thalamus being more severely affected indicating congestion. DWI: diffusion-weighted imaging.

**Figure 4:**
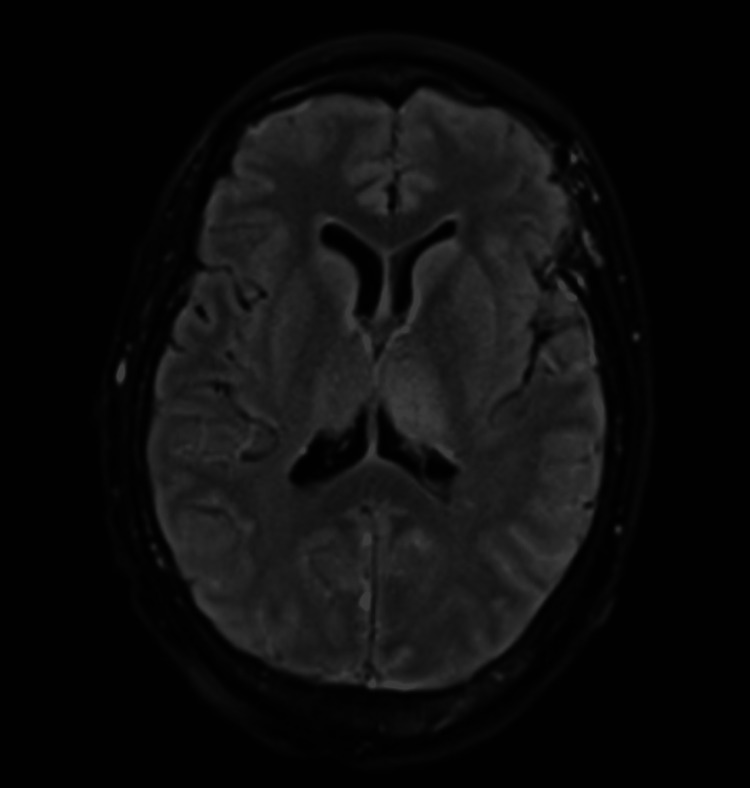
MRI of the brain FLAIR sequence in transverse view showing similar changes as noted on the DWI sequence indicating bilateral thalamic congestion. FLAIR: fluid-attenuated inversion recovery; DWI: diffusion-weighted imaging.

The patient declined antiseizure medications or evaluation with a digital subtraction angiography at that time. She presented again within three months with her typical seizure semiology, as well as prolonged altered mentation following treatment with levetiracetam. Her neurological exam, with the exception of her mentation, remained intact. A continuous electroencephalogram (EEG) was obtained revealing multifocal epileptiform discharges over bilateral frontal, left central, and right temporal regions along with left hemispheric slowing. A repeat MRI of the brain was obtained due to prolonged altered mentation and revealed an increase in signal intensity of both thalami, left more than right (Figures 3, 4). She underwent placement of a percutaneous gastrostomy tube, as she was unable to safely swallow due to her reduced wakefulness. Unfortunately, the patient’s hospital course was complicated by sepsis due to a surgical site infection and she subsequently passed away as her family transitioned her to comfort measures. Our patient’s hospitalization lasted 21 days, but we believe that her cognition was declining before her presentation. Three months prior to her acute hospitalization, she underwent the Saint Louis University Mental Status (SLUMS) examination scoring 18/30, indicating significant cognitive deficits consistent with dementia. The main domains affected were math reasoning, divergent naming and thought organization, delayed recall, and disrupted executive function. Her capacity for decision-making was considered compromised. Hence, her family was involved in making decisions for her.

## Discussion

Patients with previously undetected AVMs most frequently present with hemorrhage, while seizures are the second most frequent presentation, seen in 20-45% of cases [1]. Patients may also present with headaches or other focal neurological deficits. This case is unique, as it explores a patient with a high-grade AVM leading to bilateral thalamic congestion and prolonged decline in wakefulness and cognition.

Consciousness can be disrupted by diffuse cortical, bilateral thalamic, or reticular activating system pathologies [2]. Etiologies such as bilateral thalamic strokes (e.g., artery of Percheron), straight sinus venous thrombosis, congestion secondary to dural arterio-venous fistulas, among others, have been implicated in the development of cognitive decline and altered mentation [3,4]. Our patient's postictal state secondary to seizures alone was insufficient to account for her prolonged altered mentation. One theory we suggest is vascular congestion secondary to venous hypertension generated by this high-grade AVM leading to thalamic fiber distention [1]. Holekamp et al. and Chen et al. have described dural arteriovenous fistulas (dAVF) resulting in bithalamic congestion and progressive dementia, which improved both radiologically and clinically following the surgical intervention [3].

An alternative theory is the vascular steal phenomenon. Studies evaluating cortical plasticity utilizing functional MRI identified deficits in motor skills, verbal skills, memory, and visuospatial processing [5]. Cognitive deficits identified were associated with the hemisphere contralateral to the AVM, which can be explained by blood diversion from high to low-flow vascular systems.

Utilizing standardized forms such as the Montreal Cognitive Assessment (MOCA), the Mini-Mental State Examination (MMSE), or the SLUMS examination prior to and after the intervention may prove to be a valuable tool in assessing response to treatment after stabilization. The role of neuropsychiatric evaluation can be pivotal in evaluating those who would benefit the most from surgical intervention.

We used PubMed electronic search up to May 1, 2023, for intracranial AVM treatment, brain AVM, high-grade AVMs, and surgical management of AVMs to review the most recent literature. Dr. Hakoun, along with one of the five authors serving as a second reader, reviewed and extracted data. Inclusion criteria were studies specifying the treatments offered, duration of follow-up, and complications. Our search also included case reports or reviews published in the past 10 years. Exclusion criteria were articles in languages other than English, and papers describing other intracranial vascular malformations (e.g., dural arteriovenous fistula, cavernous malformation, and developmental venous anomaly).

The role of microsurgery

Microsurgery is considered a mainstay of AVM treatment and has had great results, particularly in AVM SM grades 1, 2, and 3. However, there is little to no evidence that the utilization of microsurgery is safe for patients with larger AVMs. Patients who suffer from larger AVMs are considered at high surgical risk and are deferred for stereotactic radiosurgical and endovascular evaluation [6]. In a retrospective review of 67 patients with ruptured cerebellar AVMs of various grades, microsurgery was utilized in five grade 4 and three grade 5 cases with reasonable results. But this review was limited by the nature of retrospective studies and only included a few patients suffering from high-grade AVMs. The patients who underwent microsurgery with a Glasgow Coma Scale score of less than 8 on presentation had worse outcomes [7].

The role of stereotactic radiosurgery

Stereotactic radiosurgery (SRS) utilization has been taking strides toward becoming the standard of care in the management of small to medium-volume AVMs. A recent meta-analysis of outcomes post-SRS for various AVM grades found seizure improvements in 74% of patients with complete remission in 60% of study participants [8]. Similar remission rates were seen in patients who underwent endovascular embolization or microsurgery. The rate of total obliteration was around 66% in 10 years [9]. The rate of hemorrhage following SRS was up to 36% in 10 years with an annual rupture risk of 2% and a potential resolution of less than 60% [9,10]. Our patient had a grade 5 AVM that received its main feeders from posterior circulation. AVMs of this caliber may be approached via multiple sessions of radiosurgery. It is important to note that the single-stage radiosurgery of a large-volume AVM either results in unacceptable radiation-related risks or low obliteration efficacy [11]. Based on the Pittsburgh Experience, some may contemplate a multi-stage SRS with 20 Gy to the majority of nidus with four weeks between stages for a total of three stages [9]. SRS may offer an opportunity for palliative and perhaps curative symptom management in patients with contraindications to surgical resection.

The role of neuroendovascular management

Endovascular embolization is used for the management of small AVMs or as adjuvant therapy for the treatment of larger AVMs. Repeatedly shown to reduce nidus size, endovascular embolization is frequently utilized as a precursor to microsurgical resection. For this reason, embolization has also been indicated prior to SRS, though some data have emerged suggesting that it may decrease the efficiency of the latter [12]. In the acute context of inoperable, ruptured AVMs, endovascular embolization has been recommended as a palliative treatment to mitigate the risk of recurrent hemorrhage. Furthermore, embolization has been indicated in instances of cerebrovascular steal as a method to alleviate vascular congestion or ischemia. Despite its supportive benefits to other treatment modalities, the impact of endovascular embolization as a primary therapy for large AVMs is uncertain with reported cure rates differing and the occurrence of recanalization unclear.

The ARUBA study, a multicenter, randomized control trial comparing surgical management of AVMs to medical treatment in patients with unruptured AVMs, found no difference in seizure outcomes [13]. However, such high-impact trials often have a limited number of patients in these high-risk groups. Based on the risk of surgical complications, we believe that maximizing medical management prior to considering surgical intervention would be ideal. However, surgical management should be considered for patients with cognitive dysfunction and refractory seizures. High-grade AVMs that involve supra and infratentorial structures are rare and further evaluation is needed to investigate the feasibility of interventions on a case-by-case basis. SRS along with adjunct endovascular embolization may be a valid option, and referring to highly specialized centers may be lifesaving. Medical management of seizures with anti-seizure medications is recommended, and the choice of medication should be made based on the patient’s clinical presentation and side effect profile. Avoiding agents known to cause mental slowing and fogginess, such as topiramate, would be preferred to avoid confabulation. Due to the rarity of these cases, measuring the outcomes of interventions may prove to be challenging.

## Conclusions

High-grade AVMs are a rare cause of bilateral thalamic congestion and subsequent dementia. Cognitive decline secondary to AVMs can go unrecognized. Hence, a thorough evaluation is necessary when encountering this patient population. Dementia can be attributed to venous hypertension or cerebrovascular steal phenomenon. The avenue of cognition should be explored when weighing the risks and benefits of treatment. Interventions such as microsurgery, SRS, and endovascular embolization should be considered in patients experiencing AVM-related dementia that is thought to be reversible. Evaluation of each case should be done by a multidisciplinary team that includes neuropsychologists, neurologists, neurosurgeons, and neuroendovascular specialists. We recommend screening patients who have clinical and radiological findings concerning AVM-related dementia with the SLUMS examination, MMSE, or MOCA, followed by a full neuropsychological evaluation prior to and following medical management and/or surgical interventions.
